# Enhanced recovery program for hip and knee replacement reduces death rate: A study of 4,500 consecutive primary hip and knee replacements

**DOI:** 10.3109/17453674.2011.618911

**Published:** 2011-11-24

**Authors:** Ajay Malviya, Kate Martin, Ian Harper, Scott D Muller, Kevin P Emmerson, Paul F Partington, Mike R Reed

**Affiliations:** Northumbria Healthcare NHS Foundation Trust, Tyne and Wear, UK

## Abstract

**Background and purpose:**

Multimodal techniques can aid early rehabilitation and discharge of patients following primary joint replacement. We hypothesized that this not only reduces the economic burden of joint replacement by reducing length of stay, but also helps in reduction of early complications.

**Patients and methods:**

We evaluated 4,500 consecutive unselected total hip replacements and total knee replacements regarding length of hospital stay, mortality, and perioperative complications. The first 3,000 underwent a traditional protocol while the other 1,500 underwent an enhanced recovery protocol involving behavioral, pharmacological, and procedural modifications.

**Results:**

There was a reduction in 30-day death rate (0.5% to 0.1%, p = 0.02) and 90-day death rate (0.8% to 0.2%, p = 0.01). The median length of stay decreased from 6 days to 3 days (p < 0.001), resulting in a saving of 5,418 bed days. Requirement for blood transfusion was reduced (23% to 9.8%, p < 0.001). There was a trend of a reduced rate of 30-day myocardial infarction (0.8% to 0.5%. p = 0 .2) and stroke (0.5% to 0.2%, p = 0.2). The 60-day deep vein thrombosis figures (0.8% to 0.6%, p = 0.5) and pulmonary embolism figures (1.2% to 1.1%, p = 0.9) were similar. Re-admission rate remained unchanged during the period of the study (4.7% to 4.8%, p = 0.8).

**Interpretation:**

This large observational study of unselected consecutive hip and knee arthroplasty patients shows a substantial reduction in death rate, reduced length of stay, and reduced transfusion requirements after the introduction of a multimodal enhanced recovery protocol.

Accelerated rehabilitation after arthroplasty may reduce morbidity and length of hospital stay, with increased satisfaction and safety after discharge (Kehlet and Wilmore 2002). Several randomized trials (Reilly et al. 2005, Andersen et al. 2007, 2008b, Larsen et al. 2008, Essving et al. 2009) have show that local anesthetic wound infiltration after total joint replacement can reduce hospital stay. A prerequisite for the success of these techniques is a multidisciplinary collaboration between patients, surgeons, anesthetists, physiotherapists, occupational therapists, and nursing staff. The Golden Jubilee Hospital in Glasgow has published results (Kinninmonth et al. 2009a, b) and their protocol has been shared with and adopted by several centers in the UK.

Endpoints, or success criteria, have uniformly been reduction in postoperative length of stay (LOS), shorter convalescence, and rapid functional recovery (Kehlet and Wilmore 2002). It is not clear, however, whether these protocols influence early morbidity and death rates after primary joint replacement. We introduced such a technique and compared its outcome against our previous results.

## Patients and methods

The “enhanced recovery” protocol was introduced in May, 2008. All patients undergoing primary hip replacement (THR) and knee replacement (TKR) under the care of 9 surgeons were included at 2 separate units within the same hospital unit. Unit 1 dealt exclusively with relatively fitter patients (ASA 1 and 2), while unit 2 dealt with all grades of ASA status because of better availability of medical and high-dependency care; thus historically, unit 2 has carried patients with a longer length of stay. The involvement of all treating staff is thought to be critical, and our team were educated en masse by early proponents of the technique (Andersen et al. 2008b, Kinninmonth et al. 2009). Minor modifications to their protocol were made to accommodate local policy. The enhanced recovery technique involves behavioral, pharmacological, and procedural modifications (Table 1). This includes the use of gabapentin, starting on the night before surgery, and tranexamic acid (15 mg/kg, slow intravenous bolus given at induction of anesthesia).

**Table 1.** Protocol followed during the different periods

Traditional	“Enhanced recovery” protocol
Generic patient and staff education.	Patient and staff education specifically detailing “enhanced recovery” principles.
General anaesthesia, spinal, or epidural according to the preference of the anesthetist and consent of the patient.	Pre-admission medication: – Gabapentin (300 mg) on the night before surgery (to continue twice daily for 5 days). – Dexamethasone—10 mg orally on the night before surgery and 4 mg intravenously at induction.
Perioperative urinary catheterization—standard intravenous fluid until next day	Perioperative urinary catheterization—as per clinical indication.
Mobilization next day.	Low-dose spinal anesthesia: – 2–3 mL of 0.25% Bupivacaine (plain) or 2 mL of 0.5% Bupivacaine (heavy). – No intrathecal opioids.
Patient-controlled opioid analgesia intravenously	Propofol intravenous infusion (0–2.5 µg/mL) ± Ketamine (0.5 mg/kg, slow intravenous bolus).
Discharge when standardized criteria were met.	Paracetamol (1 g intravenously) ± Parecoxib (40 mg intravenously). Judicious intraoperative fluid and vasopressor administration. Tranexamic acid (15 mg/kg—slow intravenous bolus at induction; withheld in cases of thromboembolic event in the last 6 months). Intra- and postoperative infiltration of local anesthetic (100 mL levobupivacaine 1.25 mg/mL). Aim for same-day mobilization. Discharge when standardized criteria met.

Anesthesis involved the use of low-dose spinal anesthesia combined with sedation, or light general anesthesia with the patient breathing spontaneously. Local anesthetic infiltration technique (Kerr and Kohan 2008) is simple, safe, cheap, and requires no special technical skill (Rostlund and Kehlet 2007). It was used in all patients after May, 2008.

Intraoperative infiltration of 80 mL 0.125% levobupivicaine using a standardized technique ensures wide-field infiltration to include joint capsule, muscles, fat, and skin. During closure, an epidural catheter is placed within the joint to exit away from the surgical field, through which a further 20 mL of levobupivicaine is infiltrated after closure. A microbiological filter is attached and this catheter is used to infuse 3 postoperative boluses of levobupivicaine 4–6 h postoperatively, again after a further 6-to 8-h interval, and lastly on the morning of day 1, before removal of the catheter.

The postoperative boluses consisted of 20 mL levobupivacaine (1.25 mg/mL) for total hip replacement and 40 mL levobupivacaine (1.25 mg/mL) for total knee replacement. The larger volume used for knee replacement was based upon the larger intraarticular space in the knee compared to the hip.

To avoid loss of local anesthetic (Nechleba et al. 2005), drains were not used in any of the patients. Knee replacements received a single wool and crepe bandage and a Cryo-Cuff (Aircast; DJO UK Ltd., Guildford, Surrey, UK) was applied to the recovery area. This has been shown to enhance and prolong analgesia (Andersen et al. 2008b).

Early postoperative mobilization started within the first 3–5 h, aiming at discharge to home once the patient was independently mobile with the help of appropriate walking aids and the standard unchanged hospital discharge criteria had been met. Patients were educated to expect some discomfort, and were required to be active participants in their recovery. Positive encouragement from all team members was considered important. Patient education is considered a key component of the enhanced recovery protocol and a common message was transmitted by each member of the team at various stages of preoperative assessment. Currently, an information DVD is provided to every patient at the time of booking for surgery.

Postoperative analgesia included gabapentin (300 mg BD for 5 days) and oxycontin (5–20 mg twice daily for 2 days) followed by tramadol (50–100 mg every 4–6 h). Post-discharge pain treatment was similar for both groups, with paracetamol, NSAIDs, and weak opioids only. Patients were reassured that despite earlier discharge they had not been abandoned, and they were all supplied with ward contact details and recommendations if they had any concerns.

Venous thromboembolism prophylaxis changed during the study period—from mechanical and aspirin to extended tinzaparin, in keeping with the evolving NICE guidance.

Hospital episode statistics (HES) on NHS patients are collected by all healthcare providers in the UK (including independent hospitals). They describe each patient episode in terms of medical diagnosis and complication codes (International Statistical Classification of Diseases and Related Health Problems (tenth revision), ICD-10 codes) and surgical procedure (Office of Population, Censuses and Surveys Classification of Surgical Operations and Procedures (fourth revision), OPCS-4 codes). Individual episode data linked to complications, which result in re-admission after a successful discharge, are included. By employing the appropriate codes, complication rates following primary THRs and TKRs can be identified. Data were requested on the incidence of mortality, return to theater (RTT) for wound problems, stroke, gastrointestinal bleeding (GIB), acute renal failure (ARF), myocardial infarction (MI), and thrombocytopenia (TCP) within 30 days of the primary procedure, and/or those who were diagnosed as having deep vein thrombosis (DVT) or pulmonary embolism (PE) within 60 days.

We present the results of the first 1,500 primary hip and knee replacement patients who went through the enhanced recovery protocol from May, 2008 to November, 2009. We compared the results with those from an unselected, consecutive series of 3,000 of our own total hip and knee replacement patients treated with traditional techniques (January, 2004 to April, 2008) immediately before the introduction of the enhanced recovery protocol.

Orthogeriatric rehabilitation is done within our Trust, and is not outsourced to another provider. Thus rehabilitation was included in the hospital stay for both groups.

Grouping of patients. Trad1: first 500; Trad2: 501–1,000; Trad3: 1,001–1,500; Trad4: 1,501–2001; Trad5: 2,001–2,500; Trad6: 2,501–3,000. ER1: first 500; ER2: 501–1,000; ER3: 1,001–1,500.

### Statistics

The two-tailed unpaired t-test was used to compare the length of stay (nights in hospital) between the 2 groups. Chi-squared tests were performed to compare the complications in the 2 groups.

## Results

There was a highly significant reduction in 30- and 90-day mortality (Table 2). The overall length of stay (LOS) decreased from a mean of 8.5 to 4.8 days and from a median of 6 to 3 days (p < 0.001). Some of the comorbidities were commoner in the enhanced recovery group (Table 3).

**Table 2.** Comparison of mortality rates between the 2 groups

	Traditional	Enhanced	p-value
	(n = 3,000)	(n = 1,500)	Chi-squared test
Death (30-day)	15 (0.5%)	1 (0.1%)	0.02
Death (90-day)	25 (0.8%)	3 (0.2%)	0.01

**Table 3.** Comparison of the demographics between the 2 groups

	Traditional	Enhanced	p-value
	(n = 3,000)	(n = 1,500)	Chi-squared test
Age (years)	69	68	
THR	1,368	630	
TKR	1,632	870	
Gender (M:F)	1,482:1,518	711:789	0.2
Comorbidities			
Hypertension	921 (31%)	673 (45%)	< 0.001
AF	143 (5%)	84 (6%)	0.2
IHD	211 (7%)	113 (8%)	0.5
IDDM	20 (1%)	18 (1%)	0.07
NIDDM	205 (7%)	150 (10%)	< 0.001
COPD	85 (3%)	67 (4%)	0.004
Alzheimer	6 (0.2%)	5 (0.3%)	0.4

AF: atrial fibrillation; IHD: ischemic heart disease; IDDM: insulin-dependent diabetes mellitus; NIDDM: non-insulin-dependent diabetis mellitus; COPD: chronic obstructive pulmonary disease.

There was a statistically non-significant reduction in myocardial and cerebrovascular complications (Table 4). Thromboembolic renal impairment requiring high-dependency care, re-admission, and return-to-theater complications remained unchanged.

**Table 4.** Comparison of complications between the 2 groups

	Traditional	Enhanced	p-value
	(n = 3,000)	(n = 1,500)	Chi-squared test
RTT (30-day)	74 (2.5%)	27 (1.8%)	0.2
Stroke (30-day)	14 (0.5%)	3 (0.2%)	0.2
GI bleeding (30-day)	18 (0.6%)	4 (0.3%)	0.1
MI (30-day)	25 (0.8%)	7 (0.5%)	0.2
ARF (30-day) **^a^**	1 (0.03%)	2 (0.13%)	0.2
DVT (60-day)	23 (0.8%)	9 (0.6%)	0.5
PE (60-day)	36 (1.2%)	17 (1.1%)	0.9
Re-admission	140 (4.7%)	72 (4.8%)	0.8

RTT: return to theater, GI: gastrointestinal, MI: myocardial infarction, ARF: acute renal failure, DVT: deep venous thrombosis, PE: pulmonary embolism

**^a^** Requiring admission to high dependency unit.

The outcome was similar for both units, with a reduction in mean LOS (p < 0.001). The mean LOS (in days) in unit 1 was higher than in unit 2, both in the traditional (unit 1: 6.6; unit 2: 9) and the enhanced recovery group (unit 1: 3.4; unit 2: 5.6), reflecting the types of patients operated in the 2 units.

**Figure 1.**
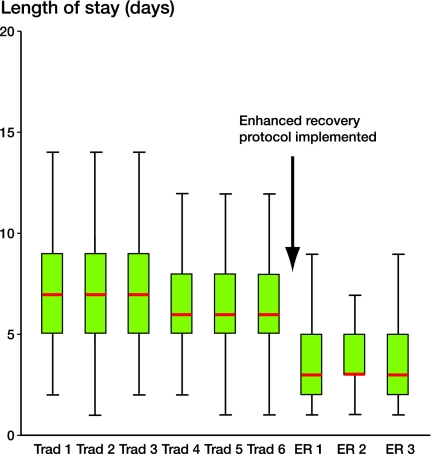
Box plot to compare the length of stay between the tradiional (Trad) and the enhanced recovery (ER) groups.

**Figure 2.**
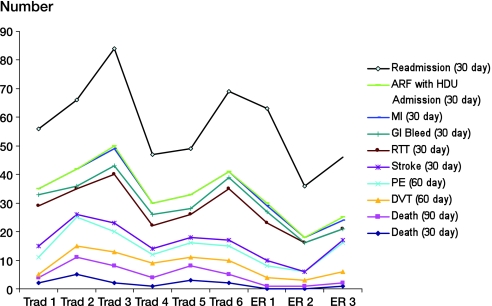
Trend of various complications through the study period. Grouping of patients is explained in the footnote to Figure 1.

The trend of length of stay over the period of the study showed a clear reduction in the median length of stay after implementation of the enhanced recovery protocol (Figure 1). There was a trend of reduction of the various complications over the period of the study (Figure 2).

The percentage of patients requiring blood transfusion decreased from 23% in 1,000 consecutive patients in the immediate pre-enhanced recovery protocol group to 9.8% in the post-enhanced recovery group (p < 0.001). There has been a uniform tranfusion policy since June, 2007; the earlier traditional patients were therefore excluded.

Preoperative dexamethasone was initially part of the protocol, but several members of the anesthetic and surgical teams were uncomfortable with the immunosuppressive properties and potential consequences of infection. It was therefore discontinued midway through the enhanced recovery study period. Subanalysis of the return-to-theater rates for wound problems revealed an RTT rate of 1.5% (7 of 453) before implementation of this change as compared to 1.9% (20 of 1,047) after it (p = 0.6).

## Discussion

The enhanced recovery concept assumes that multimodal intervention may reduce stress-induced organ dysfunction and the accompanying morbidity that results in the subsequent need for hospitalization (Kehlet and Wilmore 2002). We found a substantial decrease in mortality and some early complications following initiation of the enhanced recovery protocol in this unselected and consecutive series of 4,500 primary joint replacements performed by the same group of surgeons.

Linking of consenting patient records held in the National Joint Registry (NJR) to the corresponding records in HES has produced UK figures for 90-day mortality following primary joint replacement (National Joint Registry 2008). This includes complete national data on 102,179 total hip replacements (THRs) and 111,723 total knee replacements (TKRs) excluding patellofemoral joint replacements performed between April 1, 2003 and September 30, 2007. The data show a 90-day mortality rate following THR and TKR of 0.7% and 0.5%, respectively. Other reports quote 30-day mortality rates after primary hip and knee arthroplasty of between 0.24% and 0.85% (Fender et al. 1997, Parvizi et al. 2001a, b) and 90-day mortality rates from 0.5% to 1.1% (Seagroatt et al. 1991, National Joint Registry 2008). Before the inception of the enhanced recovery protocol, we had a 30-day mortality rate of 0.5% and a 90-day mortality rate of 0.8%, which have decreased to 0.1% (p = 0.02) and 0.2% (p = 0.01), respectively.

We have demonstrated that this technique can be adopted, and report a median reduction in length of stay of 3 days and a mean of 3.7 days. The enhanced recovery group, therefore, had 5,418 bed days less than expected. This is similar to the figures reported in other studies (Isaac et al. 2005, Reilly et al. 2005, Larsen et al. 2008). In England and Wales, more than 120,000 primary total hip and knee replacements are performed each year (National Joint Registry 2008). There is therefore a potential direct annual saving of approximately 434,520 bed days throughout the NHS. The average cost of an adult elective orthopedic bed has been estimated to be around €324 (Jones 2008). This is equivalent to an annual saving of approximately €141 million. The reduced length of stay allowed more cases to be performed per week without any additional bed capacity, and this is reflected in the shorter time period taken to reach substantial numbers in the enhanced recovery group. The cost effectiveness of joint replacement in health economic terms has been well documented (Bozic et al. 2004), and if the cost benefit of reduced complications is taken into account, there would be further potential benefit.

In a meta-analysis, intravenous tranexamic acid has been shown to be effective in reducing allogenic blood transfusion and blood loss in total hip and knee arthroplasty, without any apparent increase in the risk of thromboembolic complications such as deep vein thrombosis, pulmonary embolism, thrombotic cerebral vascular accident, or myocardial infarction (Ho and Ismail 2003). It is routinely used in this protocol, and we found a reduction in the requirement for blood transfusion from 23% in the traditional group to 9.8% in the enhanced recovery group.

A high re-admission rate may be an obvious problem with accelerated discharge (Larsen et al. 2008), with reports of 30-day re-admission rates of up to 13% (Danish National Board of Health and Assessment 2006). Our re-admission rates (traditional: 4.7%; enhanced recovery: 4.8%) remained unchanged throughout the period of the study. Our return-to-theater rate was similar to the 2% reported by Husted et al (2008).

To our knowledge, this is the largest reported observational study to have direct comparison before and after implementation of the protocol. In an unselected consecutive group of 4,500 hip and knee arthroplasty patients, we have demonstrated a highly significant reduction in death rate, reduced length of stay, and reduced transfusion requirements after the introduction of a multimodal enhanced recovery protocol.
